# Global Surgery as an emerging discipline: Why it thrives despite barriers—And what must happen next

**DOI:** 10.1371/journal.pgph.0006025

**Published:** 2026-02-20

**Authors:** Dhananjaya Sharma

**Affiliations:** Department of Surgery, Government NSCB Medical College, Jabalpur, Madhya Pradesh, INDIA; PLOS: Public Library of Science, UNITED STATES OF AMERICA

## Abstract

Global Surgery aspires to eliminate inequities in surgical care worldwide, yet it remains a field defined by paradox. This essay critically examines its conceptual, structural, and ethical flaws and why, despite the challenges, Global Surgery continues to flourish. Ten key challenges are explored, including the absence of clear definitions, a persistent awareness–action gap, unrecognized leadership from the Global South, voluntarism without remuneration, and entrenched Global North dominance in agenda-setting and authorship. These weaknesses undermine credibility, fragment initiatives, and risk perpetuating colonial legacies. Yet the field thrives—driven by idealism, moral duty, emotional rewards, interdisciplinary engagement, and growing institutional recognition. It provides fertile ground for frugal innovation, fosters vibrant peer networks, and carries a powerful moral and historical imperative. For Global Surgery to mature into a true discipline, contradictions must be reconciled through sharper definitions, equitable leadership, South–South collaboration, sustainable financing, ethical authorship, and accountability. Partnerships must move beyond symbolism toward long-term capacity building with local ownership at their core. To translate these priorities into action, this essay critically appraises the strengths and weaknesses of the emerging discipline and provides opportunities to strengthen the new discipline. To do so, it introduces PRISM framework that structures recommendations by: *Proposal*, *Relevance to Global Surgery*, *Implementation roadmap*, *Societal benefit*, and *Measurable indicators*. Ultimately, Global Surgery must evolve from a movement into a cohesive, inclusive, and accountable discipline—one that delivers on its promise of safe, timely, and equitable surgical care for all. Until then, it will embody both hope and hard truths, reflecting the tension between aspiration and reality while offering a unique opportunity to reshape the future of global health.

Global Surgery is a relatively new discipline striving to advance equitable and high-quality surgical care across global health systems, with particular attention to underserved populations in low- and middle-income countries (LMICs) [1]. It gained recognition after the publication of Lancet Commission on Global Surgery report in 2015, which provided the much needed data about unmet needs of Surgery and grabbed the World’s attention: 5 billion people not having access to safe, timely affordable surgery and anaesthesia; leading to 18.6 million preventable early deaths each year - more than the number of people who die from HIV/AIDS, malaria and tuberculosis combined; millions of patients facing catastrophic expenditures when faced with surgical costs and many low- and middle-income countries likely to lose up to 2% of GDP due to loss of proper surgical care leading to loss of productivity [2]. Despite its noble intentions, the field suffers from conceptual ambiguities, structural weaknesses, ethical dilemmas, and a troubling imbalance of power and resources between high-income and low-income settings. Yet, paradoxically, Global Surgery has captured the imagination of senior academics and young idealists alike, continues to draw support from institutions in the high and middle-income countries (HICs & LMICs) and remains a growing area of academic, policy, and clinical engagement. This essay examines the major challenges plaguing Global Surgery—and the equally powerful reasons why the field endures & even flourishes and what more needs to be done to realize its full potential.

## Part I: Ten major wrongs with Global Surgery

This section describes 10 major failings of Global Surgery.

### 1. Lack of precise definitions and metrics

Global Surgery lacks a universally accepted definition. Terms such as “access,” “equity,” and “essential surgery” are interpreted variably across studies and institutions. The oft-cited Lancet Commission metrics [2], such as the “three bellwether procedures”, serve as crude proxies but fail to capture local realities or surgical ecosystem complexity as these over-simplify the complexity of surgical ecosystems. For example:

I. Narrow scope – The three procedures (cesarean delivery, laparotomy and open fracture management) represent only a fraction of the surgical conditions encountered globally. They do not account for the wide burden of non-bellwether but highly prevalent conditions such as head and neck cancers, trauma requiring neurosurgical intervention, or non-communicable surgical needs.
II. Context insensitivity – A country’s ability to perform these procedures may not reflect the actual distribution of disease or surgical demand in its population. For instance, cesarean delivery may be a critical metric in rural setting but far less reflective of surgical capacity in urban areas where obstetric services are already widely available.
III. Systems complexity – Surgical care depends on far more than the ability to perform specific operations; it requires reliable supply chains, anesthesia, perioperative care, trained personnel, and referral networks. These broader system requirements are not captured by bellwether procedure metrics.

Thus, while valuable as a starting point for benchmarking, the bellwether procedures cannot fully represent the diversity of surgical need or the nuances of local health systems. Such definitional ambiguity hampers coordinated efforts for advocacy, research, funding, and policy implementation, despite Global Surgery’s significant visible expansion [3,4].

Additionally, measurement challenges remain central to this as surgical volume data are still incomplete due to underreporting.

### 2. A crisis of inaction despite awareness

The Lancet Commission’s wake-up call about the global burden of surgical disease still echoes all over the world: “*five billion people lack access to safe & timely surgical care*” [2]. And yet, despite the knowledge, global surgical systems in LMICs remain under-resourced. Despite inspiring global momentum, progress toward the Lancet Commission on Global Surgery’s 2030 goals has been slow and uneven, with over 160 million surgical procedures still unmet annually in the Global South [5]. The current extent of global surgical crisis is staggering—claiming lives and livelihoods at an unimaginable scale: every two seconds, someone dies from a surgically treatable condition, ~ 16 million deaths a year—a toll exceeding that of HIV, tuberculosis, and malaria combined—while nearly half the world would face financial ruin if they sought surgical care today, and 80 million are pushed into financial catastrophe solely from its cost [6]. This clearly makes the global surgical awareness-action gap one of the greatest and most preventable health crises of our time [7]. Epidemics such as COVID-19 and Ebola further overload already fragile health systems in the Global South, diverting scarce resources and widening the equity gap between those with reliable access to care and those chronically left behind. Last but not the least, recent sudden and severe funding cuts, led by the U.S. and followed by others, have further stalled progress in surgical care across LMICs [8]. These U.S.-led reductions in global health assistance—long the backbone of external support—have placed the WHO under severe financial strain and triggered cascading setbacks in LMIC surgical systems, disrupting training pipelines, supply chains, and essential services. Similar cuts to the UK’s Official Development Assistance, particularly in health systems strengthening and research, have compounded these disruptions, collectively reversing hard-won momentum in surgical equity and access. The real challenge lies in the fact that due to lack of political commitment to prioritize global surgery within health systems, national UHC budgets often allocate insufficient funds to surgical services, leaving countries overly dependent on USAID and UKAID; in health systems dominated by private hospitals and insurance schemes, this underinvestment deepens existing inequities in access to essential surgery. Very few countries address this issue through insurance schemes and policies.

### 3. Leadership qualities of Global South surgeons often go unrecognized

Surgeons from the Global South who possess contextual experience, clinical expertise, and local credibility often find themselves sidelined in favor of academics from high-income countries with stronger grant-writing or publishing track records [9]. The wisdom and innate leadership skills of these surgeons—nurtured in resource-constrained, high-volume environments—are often underrepresented in multiple domains of Global Surgery leadership—including first or senior authorship of influential papers, principal investigator (PI) status on multi-country grants, invited speaking roles at major conferences, and formal leadership positions within international organizations or consortia.

### 4. Limited and uneven training

Global Surgery suffers from a lack of institutional coherence in universities and academic medical centers in the Global South. Moreover, Faculty, even if willing, is consumed by heavy clinical workloads, leaving little scope for structured curricula or skills training in Global Surgery. Even in the Global North, dedicated departments, endowed chairs, and accredited training pathways in global surgery are still relatively rare, and that in many institutions global surgery training remains dependent on ad hoc electives and individual champions. Unlike fields such as Global Health or Infectious Diseases, which have dedicated departments, funding streams, and training programs, suboptimal teaching and training in global and rural surgery at academic institutions is a key factor contributing to inadequate preparation of trainees to meet this critical need [10].

### 5. No one gets paid: The voluntarism trap

Unlike salaried clinical or teaching duties, most Global Surgery work—whether research, teaching, or capacity-building—is undertaken voluntarily, in addition to regular responsibilities. This lack of structured compensation sends a signal that surgical equity is a charitable cause rather than a professional priority [11]. Funding grants typically cover support for Global North Principal Investigators but LMIC collaborators, students and trainees, who contribute significantly, are usually unpaid. At the grassroots level, the situation is even starker. Community health workers (CHWs), who are often the primary link between healthcare systems and underserved populations, operate with minimal stipends. Their critical work is seldom reflected in the financial structures of major Global Surgery programs; consequently, many health systems and ministries do not take Global Surgery seriously.

### 6. The allure of North-led prestige overshadowing South-driven pragmatism

In my experience—and in the view of many colleagues—hospitals in the Global South often wait for prestigious visits from Ivy League institutions, even if these visits are brief, ceremonial, and minimally impactful. Meanwhile, potentially more effective South-South collaborations are undervalued due to lack of prestige or funding [12]. Even the patients in rural areas often favor ‘foreigners’ and tend to undervalue local workers who have sacrificed city lives and careers to serve in rural communities. This skewed attention shows a colonial mindset and limits the potential for stronger, context-specific and local-innovation driven research & outcomes.

### 7. Helicopter missions are poor capacity builders

Short-term surgical camps such as plastic surgery or cataract surgery missions or “*helicopter*/ *parachute missions*” in LMICs may offer momentary relief but do little to build long-term capacity. They often involve foreign teams flying in with their own protocols and equipment, bypassing local systems. While these provide some immediate surgical relief, these missions rarely train local staff or strengthen referral and follow-up systems, resulting in minimal long-term improvement in surgical capacity [13].

### 8. Unfulfilled promises breed disillusionment

Lancet Commission on Global Surgery recommendations spurred numerous national surgical plans and memoranda of understanding across LMICs, as well as pilot programs such as WHO-led initiatives for strengthening emergency and essential surgical care. Despite these high-profile commitments, many were stalled due to lack of funding, weak local governance, or insufficient integration into national health strategies, illustrating the persistent gap between ambitious pledges and measurable implementation on the ground. This reflects a widely recognised pattern in global health in which well-intentioned declarations and high-visibility commitments often fail to translate into sustained implementation [14]. When follow-up and funding do not appear, local partners are left disillusioned and demotivated. And the Global South, scarred by decades of ‘*burnout from broken promises*’ has learned not to mistake words for action.

### 9. Inefficiency and fragmentation

Global Surgery remains a fragmented and loosely connected ecosystem, marked by overlapping programs – parallel surgical training programs or short-term missions in the same region, duplicated research - collecting overlapping data rather than coordinating or pooling resources, and competition rather than collaboration among NGOs, academic groups, and individual proponents [15,16]. Limited coordination and siloed efforts - even national surgical plans and pilot programs frequently operate in silos - working in isolation without a shared vision - waste scarce resources, dilute impact, and hinder meaningful progress on the ground. With no central governing body or unifying strategic framework, this lack of cohesion wastes resources and blunts the collective potential of the field, leaving opportunities for transformative change unrealized.

### 10. It is not really global—It is Global North-dominated

Despite its name, Global Surgery is often neither global in participation nor truly inclusive [17–20]. A few illustrative examples of this imbalance include:

I. Funding dominance – Many large global surgery grants, including those from the NIH, Wellcome Trust, or Gates Foundation, are awarded primarily to institutions in HICs, and with LMIC partners often listed only as collaborators, limiting their control over resource allocation.
II. Authorship inequities – Analyses of published Global Surgery research show that first and senior authorship is disproportionately held by researchers from HICs, even when studies are conducted entirely in LMIC settings.
III. Agenda-setting – Priority-setting workshops, conferences, and international consortia frequently have leadership dominated by HIC representatives, shaping research and policy agendas without proportionate input from LMIC clinicians who *actually* have the lived in experience of facing the local challenges.
IV. Ethical and professional concerns – This includes the need for proper local approvals rather than bypassing processes under the guise of involving a local collaborator. Reports of LMIC surgeons being excluded from key decisions, denied access to datasets, or subjected to dismissive or discriminatory behavior in multinational collaborations highlight persistent inequities and even instances of racial bias.

## Part II: Ten reasons why Global Surgery continues to thrive

Despite the litany of problems, Global Surgery continues to attract passionate professionals and students and even thrive. What explains this paradox?

### 1. Idealism remains a powerful force

For many, Global Surgery represents a moral calling. The idea of reducing unnecessary surgical deaths and disability resonates deeply with clinicians, especially young doctors seeking meaning beyond hospital walls. Many medical students and young professionals are drawn to Global Surgery because it embodies a moral and humanitarian mission. They see it as an opportunity to alleviate gross inequities in access to surgical care. For example, volunteer programs such as *Operation Smile* or *Medicines Sans Frontières* inspire thousands each year to dedicate time to patients in need, even in difficult environments. This sustained appeal of idealism helps Global Surgery recruit fresh talent continuously, often despite structural impediments, ensuring its survival and visibility in academic and policy arenas.

The essence of Global Surgery is best captured by the spirit of *Ubuntu*—the belief in our shared humanity and interdependence—reminding us that we have a collective responsibility to ensure equitable access to safe, timely, and affordable surgical care for all [21].

### 2. Noblesse oblige: The ethic of privilege

Many professionals from privileged backgrounds feel a sense of duty to “*give back*” to those less fortunate [22]. Those in HICs, benefiting from advanced training and resources, often feel a moral responsibility to share knowledge and services with less advantaged regions. Many doctors from LMICs also gravitate toward Global Surgery to serve those most vulnerable. Moreover, every nation has its own underserved communities—reminding us that Global Surgery requires no passport and is as relevant in HICs as it is in LMICs. This moral imperative fuels participation in Global Surgery work, especially when it is framed as an ethical responsibility. Such acts of perceived duty help Global Surgery retain legitimacy and support.

### 3. Feel-good hormones: The neuroscience of altruism

Engaging in acts of global service creates immediate emotional rewards & stimulates oxytocin and dopamine release. The emotional gratification of helping someone in need, often in dramatic surgical scenarios, can be profoundly motivating [23]. Global Surgery offers frequent “*highs*” in this regard and becomes addictive. Surgeons often describe Global Surgery work as “life-changing experiences” that provides an intense sense of gratification. This personal reward loop explains why many clinicians repeatedly return to volunteer work, fueling the persistence of Global Surgery as a movement.

### 4. Sense of purpose and identity

Global Surgery provides a strong sense of purpose and identity for many clinicians - a strong sense of belonging to a cause larger than themselves [24]. It allows surgeons to be more than technicians; they become advocates, educators, and change makers. This holistic professional identity is appealing, equally to both younger and older generations. For trainees, it often shapes career trajectories—many decide to pursue public health or academic roles after exposure to Global Surgery work. Conferences, online communities, and mentorship networks reinforce this identity, creating a self-sustaining culture that adds resilience to the field. Moreover, such voluntary work may also serve as an effective antidote to burnout [25]. Global surgery experiences not only expose trainees to conditions rarely encountered in the Global North but also cultivate empathic, collaborative practice, and as a means to offset declining case volume, and surgical opportunities [26].

### 5. Prestige and academic opportunities

Global Surgery is gradually earning recognition from leading institutions, special interest groups and journals, with fellowships, conferences, and publication opportunities steadily raising its profile [27]. It is now beginning to be regarded in academic circles as a serious, cutting-edge discipline; and offers a rare blend of prestige and purpose—uniting clinical practice with research, education, and advocacy to improve surgical care worldwide, especially in underserved regions. This prestige incentivizes participation, thereby keeping the field active and visible. It enables surgeons to tackle pressing global health challenges while advancing their own careers through research, teaching, and leadership.

### 6. Interdisciplinary and systems-level thinking

Global Surgery offers a broader lens to look at health systems, economics, ethics, and innovation. It allows surgeons to step outside the operating room and engage with policymakers, engineers, economists, and anthropologists to promote a sustainable future [28]. This intellectual diversity is refreshing and collaborations across these disciplines have led to several affordable surgical innovations. Moreover, such cross-pollination creates a dynamic intellectual environment that keeps Global Surgery at the forefront of global health discussions.

### 7. Recognition and support from global institutions

Endorsement from United Nations, WHO, the World Bank, and major philanthropic foundations has anchored Global Surgery within the global health agenda, lent credibility and funding to Global Surgery initiatives [29–31]. For instance, the unanimously passed resolution by World Health Assembly to “recognise surgical care as a critical and integral component of universal health coverage in 26 May 2015” went a long way towards recognition of Global Surgery as a new discipline. Such support has helped building up momentum, signals that the field matters on the world stage, and ensures funding streams & political relevance.

### 8. Opportunities for innovation and frugality

Working in resource-limited settings sparks creative problem-solving [32,33]. Global Surgery has become a fertile ground for frugal innovations—low-cost, high-impact solutions that can inform practice even in high-income countries [34]. This spirit of “*doing more with less*” appeals to innovators & problem-solvers; reinforcing the image of Global Surgery as a hub of practical and inspiring solutions. Such innovation attracts Bio-medical engineers, entrepreneurs, and policymakers alike, keeping the field vibrant.

### 9. Peer networks and collaborative energy

Global Surgery has spawned vibrant communities of practice, including young surgeon networks, journal clubs, and open-access platforms. These include *InciSioN* (International Student Surgical Network), *Global Surgery Student Alliance* in USA (GSSA), *Association of Academic Global Surgery*, *Johns Hopkins Global Surgery Journal Club*, *InciSioN Global Surgery Journal Club* and *DTC* (*aka* Global Surgery Germany) *Journal Club*. These groups organize conferences, webinars, and advocacy campaigns that engage thousands globally. The proliferation of student and trainee networks, camaraderie and shared goals energize participants; build a sense of solidarity across borders and provides constant momentum to Global Surgery.

### 10. Moral and historical imperative

As colonial legacies in global health are increasingly critiqued, Global Surgery offers a chance to “*do it better*” [35]. It is underpinned by the belief that the world must correct longstanding inequities rooted in colonial histories and systemic neglect; and this narrative continues to resonate powerfully. There is growing commitment to equity, local ownership, and mutual respect. This shift provides hope for a more ethical future.

## Part III: What needs to happen next: Translating priorities into Policy Recommendations

Priorities need to be translated in to action for Global Surgery to truly realize its true potential; hence each actionable recommendation is presented through the PRISM framework:

- P → Proposal
- R → Relevance to Global Surgery
- I → Implementation roadmap
- S → Societal benefit (tangible promises, how lives improve)
- M → Measurable indicators that show impact

### 1. Define and measure better

We need sharper definitions like the ARC-H principle (Access-limited, Resource-limited, Context-limited Healthcare) which reframes Global Surgery from the ground up, reflecting a more grounded, nuanced understanding that acknowledges where real challenges and expertise reside [36]. For the same reason, more context-sensitive metrics are needed that capture surgical equity beyond crude indicators like procedure counts [37].

Policy recommendation (PRISM):

- *Proposal:* Develop a conceptual framework like Surgical System Performance Index (surgical volume, post-operative mortality rate (POMR), access to surgery within 2-hours, catastrophic expenditure) to integrate and extend existing metrics.
- *Relevance:* It will reflect a more grounded, nuanced understanding of surgical system performance metrics enabling policy prioritization
- *Implementation:* Initial pilot in 3 LMICs countries; technical assistance for health information system integration; public release and independent audits
- *Societal benefit:* transparency drives accountability and helps target investments to underperforming regions.
- *Measure:* Timely publication of national dashboards and year-on-year improvements in key metrics.

We must establish practical, decision-relevant indicators to assess each country’s surgical system, because measures like catastrophic expenditure, while important, often fail to guide investment priorities effectively.

### 2. National Surgical, Obstetric and Anaesthesia Plans (NSOAPs) with ring-fenced financing

NSOAPs have emerged as a cornerstone for strengthening health systems, offering countries a structured framework to expand surgical capacity, equity, and quality of care. Yet without ring-fenced financing, even the most well-designed plans risk remaining aspirational blueprints rather than engines of real transformation. Country-level NSOAP frameworks and implementation experiences are documented and provide operational templates for scale-up.

Policy recommendation (PRISM):

- *Proposal:* Governments should adopt or fully operationalize NSOAPs and link them to explicit, ring-fenced budget lines within national health budgets or Universal Health Coverage benefit packages.
- *Relevance:* NSOAPs target the full surgical ecosystem (workforce, infrastructure, supply chains, governance) and therefore are surgical-system-specific instruments.
- *Implementation:* Various stages include stages: (a) rapid situation analysis and stakeholder mapping; (b) a prioritized 5-year NSOAP with costed interventions; (c) identification of funding sources (domestic, development partners, innovative finance) and formal inclusion in the national health budget; (d) annual public reporting and mid-term review.
- *Societal benefit:* Increases surgical volume, improves timely access to lifesaving care, reduces catastrophic surgical expenditure, and lowers avoidable perioperative mortality through system investments.
- *Measure:* a) Existence of ring-fenced NSOAP budget lines b) Surgical volume per 100,000 populations; proportion of facilities capable of bellwether procedures (or those chosen as more context appropriate); POMR; proportion of households reporting catastrophic expenditure from surgery.

It must be emphasized that NSOAPs are a valuable start, but without budgets, programmes, clear outcomes, and implementation strategies, they remain plans on paper—countries need fully funded National Surgical Public Health programmes to drive real system change.

### 3. Strengthen surgical data systems

If the priorities outlined above have to be translated in to reality then surgical data systems need to be strengthened.

Policy recommendation (PRISM):

- *Proposal:* Invest in surgical registries, routine data capture, and national policies that govern data ownership and equitable access.
- *Relevance:* Surgical outcomes data are essential for improvement cycles.
- *Implementation:* Fund digital registry pilots, train data stewards, and adopt national data governance policies with clear benefit-sharing clauses.
- *Societal benefit:* This will lead to evidence-based policy, improved quality and protection against exploitative data extraction.
- *Measure:* Proportion of hospitals reporting surgical data and existence of national data governance policy.

Logically, investing in infrastructure and training should come before data collection.

### 4. Normalize compensation for Global Surgery work and embed it as a discipline in institutions

Just because the goal is noble does not mean the labor should be free [11]. Fair payment acknowledges that Global Surgery is real work, not a hobby. Good intentions started Global Surgery; but only a professional model will sustain it [16].

Moreover, Global Surgery needs structured programs, funding pathways, and dedicated departments that offer career sustainability—not just volunteer prestige [38]. The educational content must be tailored to regional needs and address gaps in access and relevance. Any fragmentation wastes resources and blunts impact. With limited budgets, effective collaboration is not optional, but essential [39].

Policy recommendation (PRISM):

- *Proposal*: Universities and Ministries should create formal Global Surgery units with salaried faculty/leadership/ fellowship/ implementation scientists/ surgical policy advisors with defined responsibilities (education, research, policy support).
- *Relevance*: a) These structures will ensure sustainable progress and surgical-system strengthening will be recognized as academic and public-health work, rather than an extracurricular activity or episodic volunteerism. b) Global Surgery is professional work deserving remuneration — unpaid labour drives attrition and inequity.
- *Implementation*: Redefine faculty promotion criteria to credit Global Surgery work; create 3–5 year funded fellowships with protected time for LMIC clinicians and academics; embed Global Surgery advisors within ministry technical teams
- *Societal benefit*: This will create career pathways, reduce attrition of motivated clinicians, and ensure continuity of capacity building & policy implementation programs.
- *Measure*: Number of funded faculty positions/ fellowships awarded annually and retained in national systems after 3 years.

### 5. Center local innovations and leadership

South-based surgeons and institutions must move from token participation to true agenda-setting power [40]. Equity in leadership is non-negotiable and their wisdom must gain a prominent voice in all important matters [41]. Global Surgery system for the future must be locally-led, results-driven, productivity-enhancing and innovation-powered [42]. Global North leaders must recognise and recalibrate entrenched power asymmetries to position themselves as allies of Global South; thus empowering Global South colleagues [43–45]. Grassroots policy entrepreneurs must play a critical yet underexplored role in shaping health policy in the Global South. To harness this potential, local ideas must be prioritized over externally driven agendas and investment must be made in structured support, including training and policy institutes, to cultivate homegrown policy leadership [46]. Professor Paul Farmer’s profound advice remains ever pertinent: “*invest in people, build infrastructure, and create systems that are sustainable, not dependent*” [47].

Policy recommendation (PRISM):

- *Proposal*: Reorient funding toward LMIC-led grants; develop South–South hubs for context-specific innovations, their incubation, training and regulation.
- *Relevance*: South-based institutions must move from token roles to agenda-setting authority; as local solutions are more context-specific & sustainable. Moreover South–South exchanges promote contextually appropriate solutions rather than imported models.
- *Implementation:* Identify LMIC lead institutions and PIs; seed regional frugal-innovation centers; support local technology development, partner with local industry for manufacturing scale-up.
- *Societal benefit*: It will strengthens local research ecosystems & health economies and ensure interventions are affordable, locally appropriate and scalable.
- *Measure:* % of Global Surgery research funding awarded to LMIC Primary Investigators; number of locally manufactured frugal devices.

### 6. Move beyond performative partnerships and reimagine funding models

Short-term visits (*‘helicopter/ parachute*’ missions) and collaborations must give way to long-term, mutually respectful partnerships grounded in humility and co-ownership which must result in enhanced local capacity building [48,49]. Mercy Ships have set up a laudable model by operating hospital ships, visiting more than 600 ports across 55 + LMICs, and providing over 100,000 free, life-changing surgeries. More importantly, their model goes beyond treatment, empowering local healthcare providers through training, mentoring, and enduring partnerships, while working with host nations to strengthen surgical care systems—a truly sustainable approach [6]. Their commitment is also reflected in their published annual reports which emphasize training and local partnership as core mission components. Successful models like *Mercy Ships* and *Medicines Sans Frontières* can sustain large-scale surgical programs through robust donor-based funding models. For the same reason, funding models should be reimagined and decolonization of funding practices is a must to ensure imbalances in power dynamics are corrected [50].

Policy recommendation (PRISM):

- *Proposal*: It should be mandatory for major funders to have transparent budget shares for LMIC partners and evidence of local capacity building. All external surgical missions should be governed by MOUs specifying training outcomes, equipment handovers, and supervision plans.
- *Relevance*: Short visits create dependency and fragmentation; partnerships will deliver capacity and co-ownership.
- *Implementation*: Ensure MOUs with a minimum 40% of budget to LMIC partners; and capacity-strengthening components must be funded explicitly. Host ministries must maintain registry of external missions with an annual review of mission impact.
- Societal benefit: Improves continuity of care, follow-up and local skills retention.
- *Measure:* Proportion of external missions with MOUs and documented trainee skill improvements.

### 7. Ensure ethical authorship and data sovereignty

Publications must reflect genuine contribution, and data collected in the Global South must not be extracted without reciprocal benefit [51,52].

Policy recommendation (PRISM):

- *Proposal*: Adopt a charter endorsed by funders, journals and ministries mandating transparent authorship, local PI inclusion, mutually agreed data use agreements, and benefit-sharing clauses for datasets generated in LMICs.
- *Relevance*: Addresses systematically observed authorship and data-extraction inequities unique to global surgery collaborations.
- *Implementation*: a) Multi-stakeholder drafting inclusive of LMIC voices; (b) adopt as a funding condition by major donors; (c) journals to require charter compliance at submission.
- *Societal benefit*: Promotes equitable knowledge production, reduces *‘helicopter/ parachute research*’, and increases trust in partnerships.
- *Measure:* Proportion of funded projects with signed data-sharing agreements and local lead authorship.

### 8. Foster real accountability

Promises made under the banner of Global Surgery must be matched by transparent follow-through and mechanisms for holding actors accountable.

Policy recommendation (PRISM):

- *Proposal*: Establish independent evaluation bodies (regional or national) to evaluate NSOAP execution and publish audited dashboards linked to verified performance improvements.
- *Relevance*: Directly ties resources to surgical system outcomes and discourages performative gestures.
- *Implementation*: Define evaluation metrics; establish third-party audit contracts; create conditional disbursement mechanisms.
- *Societal benefit*: improves fund use effectiveness and ensures that investments translate into real benefits.
- *Measure:* % of financing tied to performance verification and improvement in audited metrics.

### 9. Scale open-access surgical education (e.g., SURGhub) and local language content

Every noble vision must be matched with practical steps so the ideals-practice gap is bridged [5]. Theoretically abstract goals like “surgical equity” must be translated into reality with open-access surgical education and certifiable training like SURGhub which has demonstrated rapid uptake and reach among LMIC learners [31]. However, Global Surgery education should also focus on surgical systems strengthening education, and not only on surgical skills.

Newer open-access journals such as Journal of Global Surgery One (https://jogs.one/) operate on community-supported models that charge no author fees while ensuring universal access. More such not-for-profit surgical journals should be created and sustained by national and international surgical associations, led by experts in their respective subspecialties. These bodies have a collective responsibility to guarantee free, fair, and equitable access to high-quality scientific knowledge for every surgeon, researcher, and trainee—especially those in resource-constrained settings.

Moreover, equitable partnership declarations and reflexivity statements needed now for many journals make power dynamics explicit, promote accountability, and ensure that collaborations in Global Surgery are genuinely mutual rather than extractive.

Policy recommendation (PRISM):

- *Proposal*: Governments, NGOs and academic consortia should fund open-access, low-bandwidth surgical education hubs, support translation into major regional languages, and link e-learning completion to certification and in-country mentorship.
- *Relevance*: Education tailored to the realities of low-resource surgical practice is uniquely relevant to Global Surgery
- *Implementation*: Invest in platforms, subsidize internet access for trainees, and create blended learning with local hands-on supervised training.
- *Societal benefit*: Rapid scaling of workforce competence and safe practice, especially where in-person training is constrained.
- *Measure:* Number of LMIC learners completing certified courses and correlated improvements in local Surgical System Performance Indices.

### 10. Listen to the disillusioned

Many early enthusiasts were forced to drop out due to burnout, broken promises, or disillusionment [53]. Their stories offer invaluable insights and those lessons must be learned.

Policy recommendation (PRISM):

- *Proposal*: Implement systematic exit and retention surveys, incorporate findings into fellowship design, and create psychosocial and career pathways.
- *Relevance*: Burnout and broken promises have driven valuable people away—their experience holds lessons for sustainable program design.
- *Implementation*: Establish national survey mechanism; iterate fellowship benefits; and mandate follow-up support.
- *Societal benefit*: Reducing attrition preserves skilled talent, safeguards training investments, and strengthens continuity of surgical care for communities.
- *Measure:* Rates of attrition from Global Surgery roles, reasons for exit, and improvement after policy changes.

Obviously some priorities and policy recommendations overlap, but this convergence reflects the multidimensional nature of Global Surgery and should be seen as complementary threads in a shared fabric, whose interconnected challenges demand equally interconnected solutions.

## Discussion: Reconciling hope with hard truths

Global Surgery is a field of paradoxes—aspirational yet uneven, exhilarating yet exhausting, inclusive in intent yet often exclusive in practice, visionary yet myopic—all at once. It is a space where idealism collides with realism, where professionals strive to balance care with conscience, and where dedicated practitioners often remain under-resourced. The humanitarian crisis it seeks to address is vast: an estimated 5 billion people still lack access to safe, timely, and affordable surgical care—a reality hiding in plain sight. Yet the response has too often been disjointed, top-down, and inequitable. As documented in the first part of this essay, persistent challenges remain: lack of precision in defining the field, the voluntarism trap, fragmented initiatives, superficial partnerships, and structural inequities in authorship, recognition, and funding. These are not peripheral irritants; they are foundational gaps that threaten the credibility and long-term sustainability of the discipline.

And still, Global Surgery thrives. As Part II outlined, there is something inherently magnetic about the discipline. Its vision of cross-border solidarity, its moral gravitas, its promise of meaningful change and its appeal to both logic and emotion make it a uniquely engaging field and attract both altruists and academics. It offers scope for innovation, systems-level thinking, and cross-disciplinary engagement that keeps it intellectually alive. The potential to play a transformative role in achieving global health equity is real - but only if the field reforms itself [22].

If Global Surgery is to mature as a discipline, it must recognize and embrace its own contradictions while harnessing its moral energy. As detailed in Part III, definitions must be clarified, power must be shared, and local leadership must be centered. Decolonization must move beyond tokenism to become a comprehensive restructuring of funding flows, knowledge systems, and publishing, thus ensuring equitable participation and advancing social justice [12,20]. Surgery must be recognized as a non-negotiable pillar of global health—essential to saving lives, driving economies, and deserving bold, sustained investment at every level [5]. And above all, promises must be fulfilled—not merely made.

To move from aspiration to accountability, these priorities must be translated into actionable policies. This essay therefore introduces the PRISM framework—policies seen clearly through a prism—as a structured tool for policy translation. PRISM ensures that each recommendation is articulated as a clear ***P***roposal, linked to its ***R***elevance in Global Surgery, supported by an ***I***mplementation roadmap, justified through its expected ***S***ocietal benefits, and anchored in ***M***easurable indicators of impact. By embedding this rigor, PRISM converts ideals into commitments, and commitments into outcomes.

### The tide is slowly turning: From North-Led to South-Driven

Better proxy indicators of surgical capacity, such as those outlined in DCP-3 (https://www.thelancet.com/disease-control-priorities-3), are sharpening the field’s analytical tools, while a growing recognition of the need for an authoritative, LMIC-led mechanism—akin to a new Lancet Commission chaired and authored predominantly by Global South experts—signals a long-overdue shift in who sets the agenda. Funding structures are evolving too: UK’s National Institute for Health and Care Research now mandates LMIC leadership in its global health grants (https://www.nihr.ac.uk/research-funding/global-health), and WHO Collaborating Centres in LMICs are increasingly shaping national and regional priorities. At the community level, surgical care in India, Latin America, and Sub-Saharan Africa is increasingly financed through a mix of government-funded or government-supported national health insurance schemes, public–philanthropic partnerships, multilateral aid, donor assistance, and diaspora contribution mechanisms [54]. Across several African settings, task-sharing initiatives have successfully trained large cadres of community-based surgical providers to safely perform caesarean sections, appendectomies, hernioplasty, amputations, and other life-saving procedures—demonstrating the transformative potential of this model [55,56]. Locally funded and locally led Global South training programs, grounded in contextual relevance and regional expertise, are delivering scalable improvements in surgical capacity and long-term system resilience, as highlighted by a bibliometric analysis of the 50 most influential global surgery training publications [57]. Local surgeons, long the backbone of surgical systems, continue to drive solutions that remain underreported but are finally gaining recognition alongside expanding South-led networks that assert context-grounded leadership and tackle issues still neglected—such as trauma care. At the same time, South–South and South-led innovations—from COSECSA’s training model, and Latin America’s affordable hernia-care platforms to Aravind Eye Care in India, Sri Lanka’s trauma network, and Rwanda’s Human Resources for Health reforms—demonstrate that some of the most durable progress is emerging from within LMICs. Universities such as Cape Town and UGHE are now training global surgery leadership and Masters Courses sought after even by HIC students, while Global North missions increasingly prioritize capacity-building and fund South–South collaborations. This momentum is reinforced by a renewed focus on foundational public health strategies and by Global South surgical innovation hubs (Center for Global Surgical Innovations and Low-Cost Solutions - https://www.surgicalinnovations.in/ - and Innovations in Global Surgery - https://www.surgicalinnovations.org/ - to peer networks like Pan-African Surgical Healthcare Forum & Southern African Development Community Technical Experts Working Group and Africa’s surgical colleges—that amplify locally driven agendas. Together, these developments mark a decisive shift: global surgery is becoming truly global, increasingly shaped by LMICs whose leadership, innovations, and grounded priorities have started illuminating a path the Global North itself may need to follow.

Yet even as this momentum builds, these advances remain uneven, fragmented, and insufficient to correct the deeper structural imbalances that have long constrained the field. The task ahead is not only to celebrate what is changing, but to ensure that these shifts consolidate into lasting systems reform. It is in this space—between progress made and progress still required—that the next steps for Global Surgery must be clearly defined. The Global Surgery community already possesses ample guidelines, partnership frameworks, and tools. What it has lacked is a consistent mechanism to hold itself accountable to its own aspirations. PRISM offers one pathway to bridge this gap. The Global North and South must “*walk the talk*” together—as complementary partners—dismantling colonial legacies and ensuring safe, accessible surgery for all [22]. It requires humility and sustained effort—not just good intentions or institutional prestige. It is time for Global Surgery to become truly global—not merely in geography, but in spirit, leadership, and accountability. Only when the diverse voices of Global Surgery harmonize into one clear purpose will the promise of safe, timely, and equitable care for all be realized, marking its growth from a movement into a true discipline. Until then, Global Surgery will continue to exist in a paradox—a field that struggles, yet thrives.
